# The Analysis of Biomimetic Caudal Fin Propulsion Mechanism with CFD

**DOI:** 10.1155/2020/7839049

**Published:** 2020-06-24

**Authors:** Guijie Liu, Shuikuan Liu, Yingchun Xie, Dingxin Leng, Guanghao Li

**Affiliations:** ^1^Department of Mechanical and Electrical Engineering, College of Engineering, Ocean University of China, Qingdao 266100, China; ^2^Key Laboratory of Ocean Engineering of Shandong Province, Ocean University of China, Qingdao 266100, China

## Abstract

In nature, fish not only have extraordinary ability of underwater movement but also have high mobility and flexibility. The low energy consumption and high efficiency of fish propulsive method provide a new idea for the research of bionic underwater robot and bionic propulsive technology. In this paper, the swordfish was taken as the research object, and the mechanism of the caudal fin propulsion was preliminarily explored by analyzing the flow field structure generated by the swing of caudal fin. Subsequently, the influence of the phase difference of the heaving and pitching movement, the swing amplitude of caudal fin, and Strouhal number (St number) on the propulsion performance of fish was discussed. The results demonstrated that the fish can obtain a greater propulsion force by optimizing the motion parameters of the caudal fin in a certain range. Lastly, through the mathematical model analysis of the tail of the swordfish, the producing propulsive force principle of the caudal fin and the caudal peduncle was obtained. Hence, the proposed method provided a theoretical basis for the design of a high-efficiency bionic propulsion system.

## 1. Introduction

Autonomous underwater vehicles (AUVs) are a type of marine equipment that play a significant role in improving the daily life of human beings, such as to monitor the marine environment or safeguard modern military operations. Thus, it is gradually becoming an extensive research topic both at home and abroad [1]. However, AUVs have some shortages, which greatly limit the application in the narrow, complex, and dynamic environment. For instance, the propulsive efficiency is low. Moreover, maneuvering performance and concealment are poor and they have a negative influence on environment.

In nature, fish evolved into the swimming mechanism that has an outstanding capability to produce high thrust efficiently and gains high performance in maneuvering flexibility and controllability. Recently, interest in the motion of fish has increased. A lot of attempts have been made to mimic the motion of fish and apply it to underwater vehicles and robots in the field of oceanography [2].

The most effective movements of swimming aquatic animals of almost all sizes appear to have the form of a transverse wave progressing along the body from fish head to fish tail [3], and the fishes that have faster speed are using the biomimetic caudal fin propulsion way. They have a high hydrodynamic efficiency and are applicable to long-time, long-distance swimming in this way [4]. However, the study of its hydrodynamic characteristics has not come to a unified conclusion.

Early in 1970s, Lighthill [5, 6] employed the influence of the swing of the caudal fin on the flow field according to the “slender body theory” and then came up with “large-scale slender body theory” that is more suitable for analyzing fish propulsion patterns. Until 2011, Candelier et al. [7] extended the “slender body theory” to a three-dimensional case to obtain the pressure expression and momentum expression of the slender fish body.

The above researchers made large contributions to establish and develop the “slender body theory.” There is no denying that the theory laid solid foundation for exploring the propulsion mechanism of fish. Caudal fin is one of the most important parts in fish body to generate a propulsive force. As the simplest propulsive mode of fish, caudal fin swing propulsive has been concerned by extensive researchers since the beginning of the last century. The first to study the relationship between the parameters of the caudal fin swing and the propulsive force was the “resistance hydrodynamic model” established by Taylor in 1952 [8], which was applicable to low Reynolds number.

As our country keeps a watchful eye to the marine resources, more and more scientific institutions have begun to do research work in the field of caudal fin propulsion and have made certain achievements. The influence of caudal fin stiffness [9, 10], caudal fin area [11], fin strip movement [12], and swing phase [13] on caudal fin propulsion, velocity, and efficiency has been preliminarily studied by researchers. Besides, Liu et al. [14] considered different thrusts at different frequencies and found that they had a specific optimum frequency under a specific flexible connection. Xin and Wu [15] studied the effect of the shape of caudal fin on swimming speed and efficiency in fish free propulsion and found that the shape of the optimal caudal fin varies with different swimming modes. Tomita et al. [16] clarify developmental processes of the white shark caudal fin, based on morphological observations of the caudal fin over several developmental stages.

The above researchers mainly explored the relationship between various influencing factors in theory, and some researchers have studied the advancement of fish by establishing a physical model and combining theory with experiment [17, 18]. In 2016, Yin et al. [19] took into account the thrust and resistance acting on the robot, and the thrust characteristic is an effective factor for calculating the thrust. In 2018, Zhong et al. [20] considered the interaction between the pectoral fin and the caudal fin, founding that the dynamics of the pectoral fin and the caudal fin can be used to estimate the overall swimming speed of the biomimetic fish.

At present, few studies have been carried out on the shape of caudal fin and its propulsion principle of swordfish. For the sake of bridging this research gap, in this paper, the key parameters of caudal fin were firstly calculated by using dynamic mesh technology in Fluent software. The generation of eddy currents and the generation of anti-Karman vortex streets were analyzed. Secondly, by changing the major parameters of the caudal fin, the propulsion mechanism was explored. Finally, through the study of the movement law of the fish tail, the biomimetic mechanism is fitted to the movement, which is verified to be correct and reasonable. Meanwhile, the mechanism of the propeller force during the propulsion process is further explored. By using the linkage, the caudal peduncle was fitted by a bionic method, which laid the foundation for the underwater robots to realize high-efficiency propulsion.

## 2. Kinematic Modelling Based on CFD

### 2.1. Establishment of a Finite Element Model

**Figure 1:**
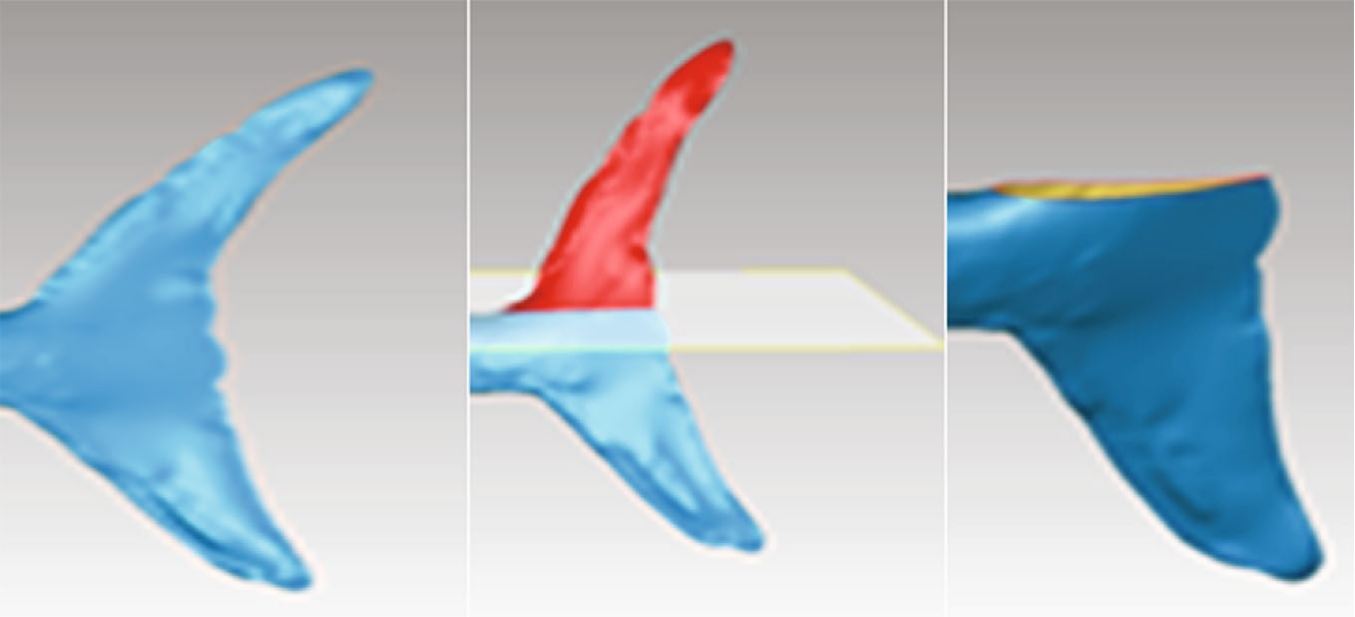
Data model of caudal fin.

The data of caudal fin was collected by 3D scanning and reverse engineering. Then, a three-dimensional model was imported into Solidworks software. In the software, the transverse symmetry surface of the caudal fin was sliced to obtain the transverse interface of the caudal fin. The two-dimensional calculation model of the caudal fin is shown in Figure 1.

**Figure 2:**
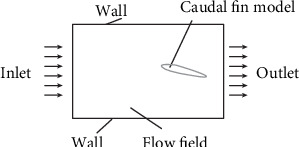
Calculation domain setting.

The CFD calculation domain setting model is displayed in Figure 2. The flow field was established to be 1800 mm × 600 mm. The caudal fin has a length of 150 mm and a maximum thickness of 15 mm. The outer rectangular border is the flow field boundary. In order to ensure accuracy and control the number of meshes, a sufficient number of nodes was arranged on the profile of caudal fin to encrypt the meshes near the caudal fin, and the meshes for the flow boundary were less relative.

**Figure 3:**
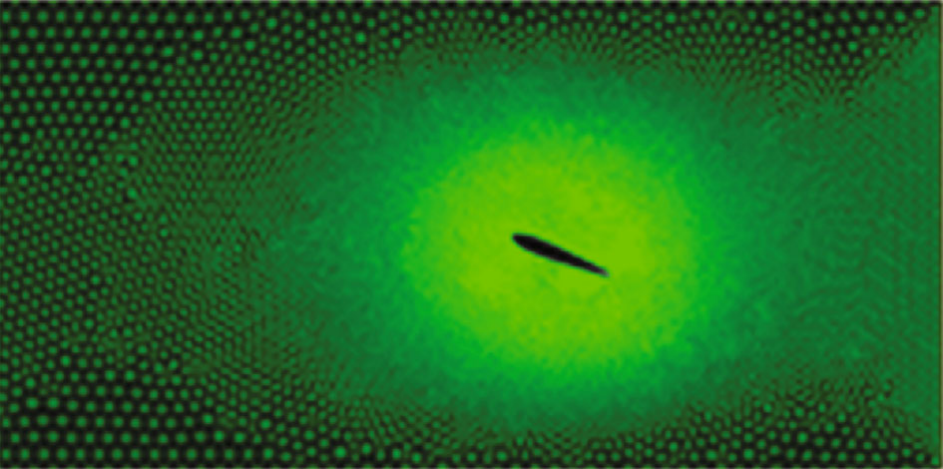
Finite element model.

The boundary conditions are exhibited in Table 1, and the movement of the caudal fin model was controlled by a UDF function. The mesh model is shown in Figure 3, and the number of grids was about 31652.

### 2.2. The Motion of Fish Body and Function of Fish Body Wave

**Figure 4:**
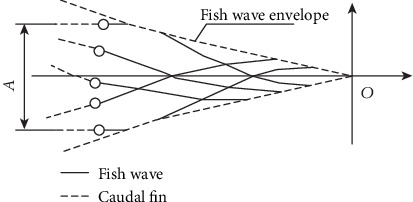
Fish body wave and fish amplitude envelope.

In the process of propulsion, the fish mainly relies on the fluctuations of the spine curve to generate a propulsive force. Through extensive biological observations and experimental studies of fish behavior, researchers have found that an implicit traveling wave is in the propulsive motion generated by the swinging caudal fin and the flexible body, which travels from the posterior neck to the tail. The bending of the spine and muscle tissue makes the fish appear wavy morphology, and the amplitude is gradually bigger from the fish head to the fish tail. The wave velocity of the traveling wave also known as “fish body wave” is greater than the forward speed of the fish body. The corresponding mathematical function expression is called the fish body wave function. To some extent, the fish body wave function can be seen as synthesized by the fish wave envelope and sinusoidal curve, as shown in Figure 4.

The wave function of the fish body begins from the center of the inertia force of the fish body and gradually extends to the caudal peduncle, and its curve equation [21] can be expressed as

(1) $$\begin{array}{c} y_{\text{body}}\left(x,t\right)=\left(c_{1}x+c_{2}x^{2}\right)\sin\left(kx+\omega t\right), \end{array}$$

where *y*_body_ is the lateral displacement of the fish, *x* is the axial displacement of the fish, *k* is the multiple of wavelength (*k* = 2*π*/*λ*), *λ* is the wavelength of the fish body wave, *c*_1_*x* + *c*_2_*x*^2^ is the fish wave amplitude envelope function, *c*_1_ is the primary coefficient of the fish body wave amplitude envelope, *c*_2_ is the quadratic coefficient of the fish body wave amplitude envelope, and *ω* is the fish body wave frequency (*ω* = 2*πf*).

The swing amplitude of the caudal fin and the distribution of the body wave amplitude can be adjusted by adjusting the value of *c*_1_ and *c*_2_.

### 2.3. Main Parameters of Hydrodynamic Performance of Caudal Fin

The St number is a parameter that expresses the characteristics of the wake structure [22]. It indicates the frequency of the swirl and the distance between them. For the fluctuating caudal fin, the St number is calculated by the following formula:

(2) $$\begin{array}{c} \text{St}=\frac{fA}{V}, \end{array}$$

where *f* represents the swing frequency of caudal fin (Hz), *A* represents the caudal fin heaving motion amplitude, and *V* is the average swimming velocity.

The angle of attack *δ*_max_ is defined as when the fins pass the equilibrium position, the angle moves between the tangential direction of the propulsive wave and the axis of symmetry of the caudal fin, which can be expressed as

(3) $$\begin{array}{c} \delta_{\max}=\phi -\theta_{0}, \end{array}$$

where *ϕ* indicates the angle between the *X*-axis and the tangential direction of the propulsive wave and *θ*_0_ indicates the angle between the geometric axis of symmetry and the *X*-axis when the tail fin passes the equilibrium position.

### 2.4. Basic Equation Based on CFD Numerical Calculation

CFD is a numerical calculation method for solving flow control equations [23]. Considering viscous and incompressible flow, the following continuity equation and motion equation are established.

(4) $$\begin{array}{c} \frac{\partial \rho }{\partial t}+\frac{\partial }{\partial x_{i}}\left(\rho u_{i}\right)=0, \end{array}$$

(5) $$\begin{array}{c} \frac{\partial }{\partial t}\left(\rho u_{i}\right)+\frac{\partial \left(\rho u_{i}u_{j}\right)}{\partial x_{j}}=-\frac{\partial p}{\partial x_{i}}+\frac{\partial p}{\partial x_{j}}\left(\mu \frac{\partial u_{i}}{\partial z_{j}}-\rho \bar{{u_{i}}^{\prime }{u_{j}}^{\prime }}\right)+S_{i}, \end{array}$$

where *ρ* represents the density of fluid, *t* represents the time, *u*_*i*_ represents the velocity of fluid, *x* is the space coordinates, *p* represents the fluid pressure, *μ* represents the kinematic viscosity coefficient, and *S*_*i*_ represents the user-defined source term.

In order to solve Equations (4) and (5), it is also necessary to add a turbulent transport equation. It has been calculated that the Reynolds number of all the working conditions is between 4 × 10^4^ and 1.4 × 10^5^, so the standard *k*‐*ε* [24, 25] model is used for calculation. It has been verified that the standard *k*‐*ε* model is suitable for practical engineering flow calculations because of its high robustness and reasonable accuracy. For further solving the equations above, the coupled implicit algorithm is utilized; hence, variables such as pressure, velocity, and stress can be obtained simultaneously [26].

## 3. Hydrodynamic Calculation

### 3.1. Analysis of the Mechanism of Caudal Fin Propulsion

The fluctuation frequency *f* = 0.8 Hz, the swing amplitude *A* = 120 mm, the fluctuation period *T* = 1.25 s, and the phase difference of the heaving and pitching movement 60° are selected for calculation [5, 13, 27].

**Figure 5:**
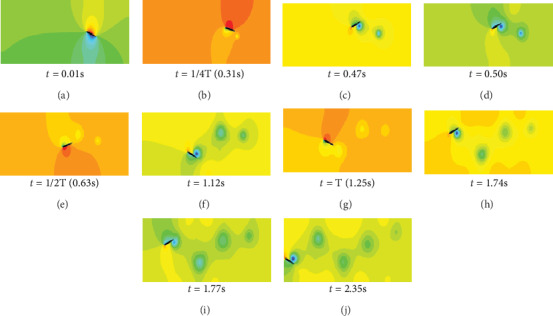
The fluid pressure distribution of the fluctuation caudal fin at different times.

As shown in Figure 5, when *t* = 0.01 s, the caudal fin begins to move forward. At this time, caudal fin is around in anticlockwise rotation, and the upper side of the caudal fin fluid pressure gradually increased. Meanwhile, the lower side of the pressure becomes smaller due to the formation of a low-pressure area.

When *t* = 1/4 T (0.31 s), the caudal fin reaches to the highest position of the swing. The lower side of the fish tail forms a low-pressure zone, and the swirl current is generated by the front end of the caudal fin, which is the beginning of the second swirl. In *t* = 0.5 s, the rotation of the swirl direction is counterclockwise rotation, which is opposite to the first swirl direction.

When *t* = 1/2 T (0.63 s), the second swirl completely falls off and the high- and low-pressure zones on both sides of the caudal fin appear mutative. The lower side is the high-pressure area, and the upper side is the low-pressure area.

*t* = 1.74 s and *t* = 1.77 s are the fourth swirl belonging to the second cycle, and mechanism is similar with the second swirl. *t* = 2.35 s is the fifth swirl, and it also belongs to the second cycle, the mechanism of which is similar with the third swirl.

After the analysis of the caudal fin swimming process, we can acquire that the caudal fin swims in a wave manner. On the upper and lower sides of the caudal fin, the high-pressure and low-pressure regions are formed according to the pitching direction. The forward swirl is gradually formed at the front end of the caudal fin. The swirl of the body becomes larger as the caudal fin swings. Meanwhile, the swirl moves toward the end of the caudal fin, and it finally falls off. By observing the direction of rotation of the five shedding swirls, it can be found that the first, third, and fifth swirls are below the *X*-axis and the direction is clockwise. The second and fourth swirls are above the *X*-axis, and the direction of rotation is clockwise. It can be found that these five shedding swirls are in the tangential direction of *X*-axis and opposite to the swimming direction of the caudal fin. And then the Karman vortex shedding is formed, forming a backward jet to result in forward thrust.

### 3.2. Effects of St Number

The operating condition is selected with the swing amplitude *A* = 120 mm, and the phase difference is 60°.

**Figure 6:**
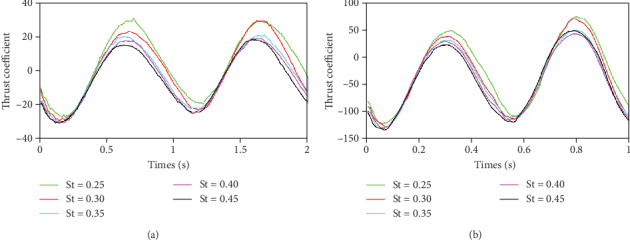
Forced condition of different values of St: (a) *f* = 0.5 Hz; (b) *f* = 1 Hz.

As depicted in Figure 6, it can be seen that when the frequency is constant, as the St coefficient increases, the flow velocity decreases, and the magnitude of the thrust coefficient gradually becomes smaller. The lower limit of the thrust coefficient is substantially the same under any working condition because the upper limit of the thrust coefficient decreases as the St coefficient increases. When the frequency is 1 Hz, it can be seen that although the magnitude of the thrust coefficient changes with the change of the St number, the amplitude of the upper limit changes significantly. At the same time, as the frequency increases, the lower limit of the thrust coefficient also increases.

Since the caudal fin moves in the negative direction of the *X*-axis, the negative value in Figure 6 indicates the same propulsive force as the caudal fin move direction, and the positive value indicates the resistance.

**Figure 7:**
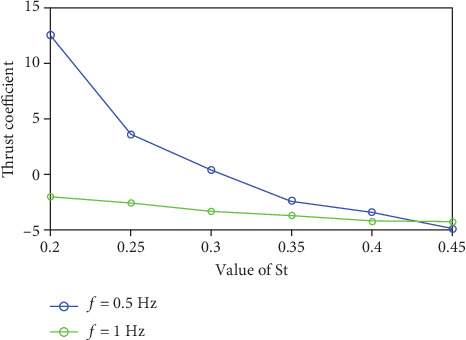
The mean value of the force of the caudal fin changes with St number.

It can be seen from Figure 7 that the variation law of the average force curve is gradually increasing with the increase of the St number, but the range of the increasing amplitude is gradually smaller. When St = 0.25, the force generated by the fins at *f* = 0.5 Hz is not conducive to the advancement of the fish body. It can be obtained that the swing frequency has a huge influence on the thrust coefficient. The fish body can overcome the flow resistance by adjusting its own tail-end frequency in time according to water velocity to avoid the force of the caudal fin to hinder the movement.

### 3.3. Effects of Phase Difference and the Angle of Attack

During the process of fluctuation of the caudal fin, there is a phase difference between the heaving and pitching movement. The different motions can be obtained through changing the phase differences and then the hydrodynamic numerical simulation can be analyzed, respectively.

**Figure 8:**
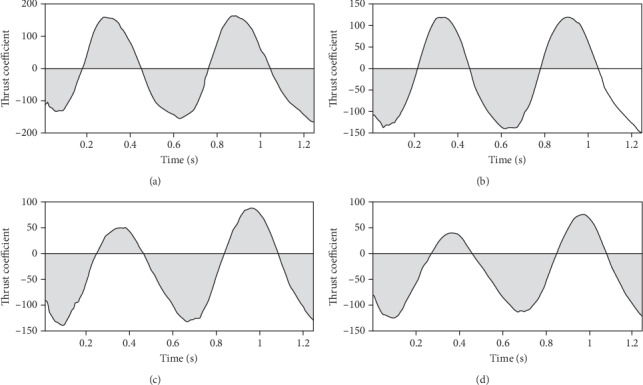
Resistance coefficient versus time in a cycle under different phase difference. (a) Phase difference is 30°, angle of attack is 31.7°; (b) phase difference is 40°, angle of attack is 28.4°; (c) phase difference is 50°, angle of attack is 25.6°; (d) phase difference is 60°, angle of attack is 21.6°.

The thrust coefficient changing with times is shown in Figure 8. It can be obtained that the force of the caudal fin is the same as the direction of advancement at the beginning. The direction of force changes with the heaving and pitching movement, which becomes the opposite direction with the fish swimming, and is not conducive to fish for forward. In Figure 8, the shaded portion below the line of *y* = 0 indicates that it is conducive to fish for moving forward while the shaded portion above the straight line of *y* = 0 indicates that it is not conducive to fish for moving forward. The longer time it takes to promote the advancing force of the caudal fin, the more favorable to forward. Comparing Figures 8(a)–8(d), it can be seen that as the increase of the phase difference, the larger the shadow area below the *y* = 0 line, which means the longer time to propulsion in a cycle.

**Figure 9:**
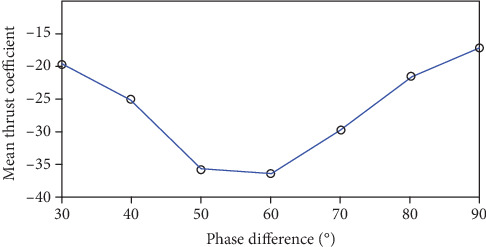
Mean thrust coefficient diagram of caudal fin under different phase difference.

As displayed in Figure 9, when the phase difference is 50-60° (the angle of attack is 21.6-25.6°), the caudal fin is propelled by a large force, so it is a relatively optimized mode of motion in this condition.

### 3.4. Effects of Swing Amplitude

**Figure 10:**
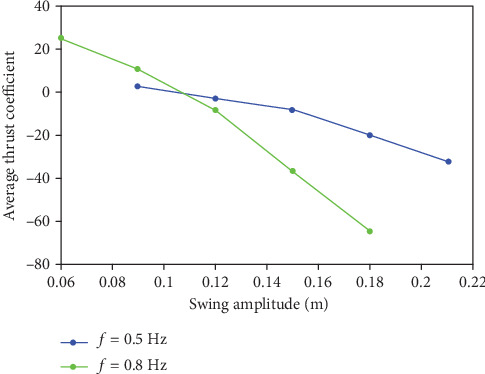
Comparison of average thrust coefficient in different swing amplitude.

Based on above analysis, the following parameters are selected for calculation: the phase difference is 60°, and the angle of attack is 23.3. The swing amplitude *A* is selected as the following values: *A* = 90 mm, 120 mm, 150 mm, 180 mm, and 210 mm. In order to obtain the relationship between the thrust coefficient and the swing amplitude intuitively, Figure 10 was drawn.

As shown in Figure 10, it can be seen that as the swing amplitude increases, the propulsion force of the caudal fin in the swimming direction increases. When the swing frequency is *f* = 0.8 Hz and the swing amplitude is 90 mm and 120 mm, the swing of the caudal fin will generate a force that hinders the advancement of the fish body. When the swing amplitude is larger than 150 mm, the propulsive force for promoting motion will be generated. In addition, comparing the two curves, it can be seen that when the swing amplitude is 180 mm and 210 mm, a slightly higher frequency can provide a more effective propulsion force for the caudal fin.

### 3.5. Study on Propulsion Performance of Double Caudal Fins in Flow Field

In many cases, the fish do not swim alone. The caudal fin swing of the former fish will cause the flow field to produce a certain regular wake vortex. The analysis of the way of the caudal fin using the wake vortex energy will be conducive to provide the development of basic theory for underwater biomimetic propulsion.

Swing frequency, swing amplitude, the phase difference, and the distance between two caudal fins will affect the mutual vortices in the flow field. Among these parameters, the effect of the two caudal fins' angle of attack on the wake vortex is mainly studied. In the process of group swimming, the swimming gait of fish is basically similar. So, we mainly changed the angle of attack during the hydrodynamic numerical simulation analysis, and other parameters are set to the same.

#### 3.5.1. Simulation Model Establishment

**Figure 11:**
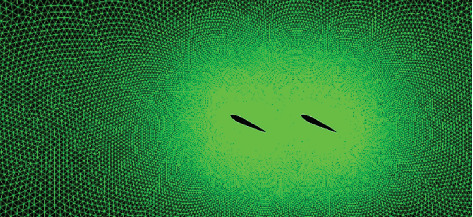
Calculation domain creation and meshing.

The flow field is established with a length of 2300 mm and a width of 1000 mm. The distance between the two caudal fins is 250 mm. The caudal fin model and boundary conditions are set to the same as before. The calculation domain creation and meshing are shown in Figure 11.

#### 3.5.2. Wake Vortex of Double Caudal Fins

**Figure 12:**
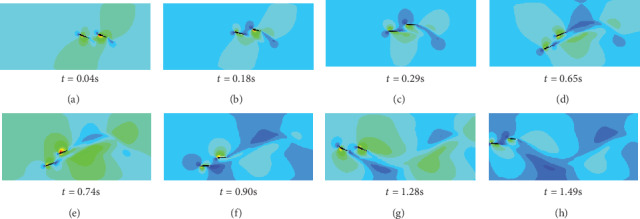
Wake vortex of double caudal fin pressure cloud.

In Figure 12, the double swinging caudal fins have the same swing amplitude in *A* = 150 mm. The swing frequency is *f* = 0.8 Hz. And the heaving and pitching movement phase difference is 60°. At *t* = 0.04 s, the double caudal fins started to move, and the trailing edges of the double caudal fins began to form eddy currents. From *t* = 0.18 s to *t* = 0.29 s, the double caudal fins swing simultaneously, and the two vortices formed at the right rear of the double caudal fins gradually converge into a large vortex. Double swinging caudal fins simultaneously sway at *t* = 0.65 s to *t* = 0.90 s and form the upper and lower vortices at the trailing edge of caudal fin. At *t* = 1.28 s, two rows of eddy currents are formed on the lower two sides of the swimming track. Due to the proper spacing and the same swing parameters during the entire swing, the rear caudal fin can add its own vortices to others without destroying the wake vortex of front caudal fin. The superimposed vortex will be beneficial to the rear caudal fins to produce more efficient propulsion.

#### 3.5.3. Double Caudal Fin Motion Tail Vortex Dissipation Mode

**Figure 13:**
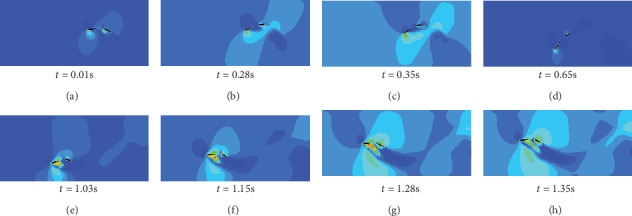
Vortex's dissipation model pressure cloud.

As shown in Figure 13, when *t* = 0.01 s, the double caudal fins just started to swing and a wake vortex was formed at the trailing edge position of the double caudal fins. Between *t* = 0.28 s and *t* = 0.65 s, the rear caudal fin swayed with the front one and destroyed the wake vortex formed during the swinging of the front caudal fin. During the latter half of the caudal fin motion (between *t* = 1.03 s and *t* = 1.35 s), the wake vortex caused by the swing of the rear caudal fin failed to overlap with the eddy current of the front caudal fin. Due to the difference of angle of attack between double caudal fins, the rear caudal fin will destroy the vortex produced by the front caudal fin, resulting in vortex dissipation that is not conducive to the effective advancement of the rear caudal fin.

## 4. The Control Method of Tail Movement

### 4.1. The Analysis of Tail Movement

The biomimetic tail propulsion mechanism mainly includes the caudal peduncle and the caudal fins. The movement of the caudal fin is driven by the caudal peduncle.

#### 4.1.1. Caudal Fin Simplified Model

**Figure 14:**
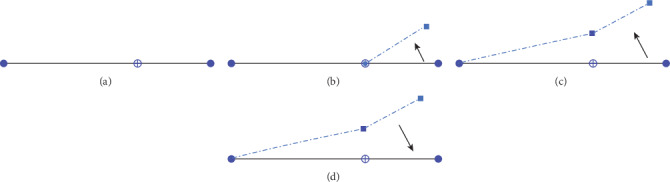
Tail swing diagram.

When biomimetic underwater vehicle is in the process of swimming, the process is shown in Figure 14. Firstly, the static state is shown in Figure 14(a). The caudal fin in the quiescent state does not occur angular swing. Then, as shown in Figure 14(b), the caudal peduncle does not occur swing and the caudal fin begins to swing upward. Subsequently, the caudal peduncle and the caudal fin swing together and the caudal peduncle swings in a large angle as shown in Figure 14(c). Lastly, as shown in Figure 14(d), the caudal peduncle and the caudal fin swing to the initial position from the maximum swing angle. After that, they swing from the equilibrium position to the opposite direction.

#### 4.1.2. Tail Movement Model Establishment

The main part of the tail movement part includes the caudal peduncle movement and the caudal fin movement.

In the study of fish tail movement, the caudal fin movement is simplified as a rigid hydrofoil moving in the uniform flow field. The way of movement is around itself doing pitching and heaving swing compound movement. The equation of motion is expressed as follows:

(6) $$\begin{array}{c} \left\{\begin{array}{c} \text{y}=\text{A}\times \sin\left(2\pi \text{ft}\right),\\ \theta_{1}=\theta_{0}\times \sin\left(2\pi \text{ft}‐\varphi \right). \end{array}\right. \end{array}$$

We can get the rising-sinking speed along with the *Y*-axis of caudal fin and the pitching angular velocity around the *Z*-axis through the derivation of formula (6):

(7) $$\begin{array}{c} {\text{V}}_{1}=2\pi \text{fA}\times \cos\left(2\pi \text{ft}\right), \end{array}$$

(8) $$\begin{array}{c} \omega =2\pi f\theta_{0}\cos\left(2\pi ft-\varphi \right). \end{array}$$

The half of caudal fin expansion is *r*, so the caudal fin swing speed is

(9) $$\begin{array}{c} V=\sqrt{{V_{1}}^{2}+{\left(\omega r\right)}^{2}+2{\text{V}}_{1}\omega r\cos\theta_{1}}. \end{array}$$

Then, we can get the speed of the caudal fin relative to the fluid:

(10) $$\begin{array}{c} {\vec{V}}_{1t}={\vec{V}}_{0}+\vec{V}, \end{array}$$

where *V*_0_ indicates the flow velocity and *V* indicates the moving speed of caudal fin along the *Y*-axis.

As there is a phase difference *ϕ* between the caudal peduncle and the caudal fin when they swing, the swing amplitude of caudal peduncle is set as *A*_0_, so the swing law of caudal peduncle can be expressed as

(11) $$\begin{array}{c} \theta_{2}\left(\text{t}\right)=\theta_{0}\times \sin\left(2\pi \text{ft}‐\phi \right). \end{array}$$

The swing angular velocity of the caudal peduncle can be expressed by the equation after the derivation of the above formula:

(12) $$\begin{array}{c} \omega_{2}\left(\text{t}\right)=2\pi \text{f}\theta_{0}\times \cos\left(2\pi \text{ft}‐\phi \right). \end{array}$$

**Figure 15:**
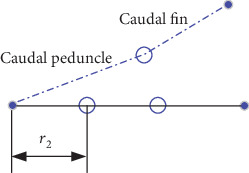
Schematic diagram of caudal peduncle swing.

According to the theory of the wave plate [28], the caudal peduncle movement model can be simplified as a rigid plate. The swing speed of the caudal peduncle can be approximated by the linear velocity of the center of gravity of the caudal peduncle. As shown in Figure 15, the distance of caudal peduncle to the fish swing joints is set as *r*_2_.

When the caudal peduncle is moving in accordance with sinusoidal law, the displacement of the center of gravity can be approximated represented by the following formula:

(13) $$\begin{array}{c} x_{2}=r_{2}\times \sin\theta_{2}=r_{2}\times \left(\theta_{0}\times \sin\left(2\pi \text{ft}‐\phi \right)\right). \end{array}$$

The speed of the caudal peduncle center of gravity can be expressed as

(14) $$\begin{array}{c} {\text{V}}_{2}\left(\text{t}\right)=2\pi \text{f}r_{2}\times \theta_{0}\times \cos\left(2\pi \text{ft}‐\phi \right). \end{array}$$

Flow velocity is set as *V*_0_, so that the relative velocity of the center point of the caudal peduncle is

(15) $$\begin{array}{c} {\vec{V}}_{3}\left(t\right)={\vec{V}}_{0}+{\vec{V}}_{2}\left(t\right). \end{array}$$

### 4.2. Establishment of a Kinematic Model of Tail Motion

**Figure 16:**
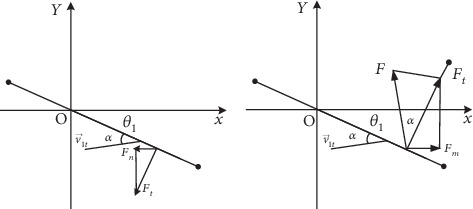
Schematic diagram of the caudal fin's fluid pressure decomposition and lift force analysis.

When the fish is moving in the flow field, the tail will be subjected to the pressure of the fluid from all directions. The fluid pressure on the surface of the caudal fin is set as *F*_*t*_. According to the Bernoulli principle, the analysis of force is as shown in Figure 16.

The propulsive force generated by fluid pressure:

(16) Ft1=12ρV1t2S1sinθ1=12ρSV02+4π2f2A2cos22πft+ω2r2+2V1ωrcosθ11sinθ1.

According to the wing theory [29, 30], the lift effect on caudal fin is set as *F*; the stress analysis is shown in Figure 16.

The lift force of the fluid on the caudal fin in the vertical direction of the caudal fin is

(17) $$\begin{array}{c} F_{\text{s}}=2\pi \rho {LCV}_{1\text{t}}^{2}\sin\alpha \cos\alpha , \end{array}$$

where *α* is the instantaneous relative angle of attack of the caudal fin;

(18) $$\begin{array}{c} \alpha =\text{arctg}\left(\frac{V_{1\text{t}}}{V_{0}}+\theta_{1}\right)=\text{arctg}\left(\frac{4\pi^{2}f^{2}A^{2}\cos^{2}\left(2\pi ft\right)+\omega^{2}r^{2}+2V_{1}\omega r\cos\theta_{1}}{V_{0}}+\theta_{1}\right), \end{array}$$

where *θ*_1_ is the instantaneous swing angle of the caudal fin, *L* is the length of slender, and *C* is the chord length.

The driving force produced by the lifting of the caudal fin is

(19) $$\begin{array}{c} F_{s1}=2\pi \rho {LCV}_{1\text{t}}^{2}\sin\alpha \cos\alpha \sin\theta_{1}=2\pi \rho LC\left(V_{0}^{2}+4\pi^{2}f^{2}A^{2}\cos^{2}\left(2\pi ft\right)+\omega^{2}r^{2}+2V_{1}\omega r\cos\theta_{1}\right)\sin\alpha \cos\alpha \sin\left(\theta_{0}\times \sin\left(2\pi ft‐\varphi \right)\right). \end{array}$$

From the above analysis, we can obtain the total propulsion produced by the moving caudal fin:

(20) F1=Ft1+Fs1=12ρSV02+4π2f2A2cos22πft+ω2r2+2V1ωrcosθ11sinθ1+2πρLCV02+4π2f2A2cos22πft+ω2r2+2V1ωrcosθ1sinαcosαsinθ0×sin2πft‐φ.

The analysis and calculation of the propulsion is similar to that of the caudal fin. The total propulsion calculation method of the caudal peduncle can be expressed as follows:

(21) $$\begin{array}{c} F_{2}=\frac{1}{2}\rho \left(V_{0}^{2}+4\pi^{2}f^{2}{r_{2}}^{2}\times \theta_{0}^{2}\times \cos^{2}\left(2\pi ft‐\phi \right)\right)\times S_{2}\times \sin\theta_{2}+2\pi \rho LC\left({V^{2}}_{0}+4\pi^{2}f^{2}{r_{2}}^{2}\times \theta_{0}^{2}\times \cos^{2}\left(2\pi ft‐\phi \right)\right)\sin\alpha_{2}\cos\alpha_{2}\sin\left(\theta_{0}\times \sin\left(2\pi ft‐\phi \right)\right). \end{array}$$

### 4.3. Gait Fitting of Fish Body Tail Biomimetic Mechanism

In Section 4, we used the link mechanism to simulate the fish body wave, so it is necessary to control the swing angle of each joint in order that each connection endpoint can approximately fit the fish wave curve. The *λ* is defined as the ratio of the length of the tail swing to the whole wavelength. It is essential to ensure that each linkage is continuous and the end point is at the end of the last linkage on the fish body wave curve. The end position of linkage satisfies the following equations:

(22) $$\begin{array}{c} \left\{\begin{array}{c} {\left(x_{i,j}-x_{i,j-1}\right)}^{2}+\left(y_{i,j}-y_{i,j-1}\right)={L^{2}}_{j},\\ y_{i,j}\left(x,t\right)=\left(c_{1}x_{i}+c_{2}{x_{i}}^{2}\right)\sin\left({kx}_{i}-\frac{2\pi }{M}i\right), \end{array}\right. \end{array}$$

where (*x*_*i*,*j*_, *y*_*i*,*j*_) is the angular coordinate of the “*j*” linkage at the moment *i* in the swing period, *x*_*i*,*o*_ = 0, *x*_*i*,5_ = *λ* · 2*π*, 1 ≤ *j* ≤ 5, 0 ≤ *i* ≤ *M*.

Bring *L*_*j*_ into the formula (23), we can solve the coordinates of each endpoint at *i* = 0, *i* = 1 until *i* = *M*. After that, we can get the angle *θ*_*i*,*j*_ between each linkage and fish body, which can be expressed as

(23) $$\begin{array}{c} \theta_{i,j}=\text{artan}\left[\frac{x_{i,j}-x_{i,j-1}}{y_{i,j}-y_{i,j-1}}\right]. \end{array}$$

The swing angles of the five steering gears are calculated, respectively,

(24) $$\begin{array}{c} \left\{\begin{array}{c} \varphi_{1}=\arctan\left(\frac{y_{1}-y_{0}}{x_{1}-x_{0}}\right),\\ \varphi_{2}=\arctan\left(\frac{y_{2}-y_{1}}{x_{2}-x_{1}}\right)-\varphi_{1},\\ \varphi_{3}=\arctan\left(\frac{y_{3}-y_{2}}{x_{3}-x_{2}}\right)-\varphi_{2},\\ \varphi_{4}=\arctan\left(\frac{y_{4}-y_{3}}{x_{4}-x_{3}}\right)-\varphi_{3},\\ \varphi_{5}=\arctan\left(\frac{y_{4}-y_{3}}{x_{4}-x_{3}}\right)-\varphi_{4}. \end{array}\right. \end{array}$$

Through the analysis of the fish body wave function, we can obtain that as the number of joints increases, it is easier to fit the fish body wave curve, but it is more difficult to coordinate control between the steering gear (the motion of the tail swing is controlled by the steering gear). Thus, the endpoints of the five joints should be fitted to the corresponding fish body wave curve as much as possible. More importantly, the working angle of each steering gear and their mutual position relationship should be well controlled. In this way, the bionic underwater vehicle can be moved like a fish, thus improving the propulsion efficiency and saving energy.

## 5. Conclusion

In this paper, the fast-moving swordfish was taken as the research object to explore the flow field structure of the swordfish caudal fin swinging. The mechanism of the caudal fin propulsion was preliminarily investigated, and the bionic mechanism motion fitting was carried out. The main conclusions were obtained as follows:

1. During the swinging process, the fish-tail forms a wake vortex due to the transformation of the high-pressure and low-pressure zones. The tangential direction of the body vortex at the *X*-axis position is opposite to the swimming direction of the caudal fin, thereby forming a backward jet in the tail flow field, which produces a forward thrust
2. When St number is in the range of 0.25 to 0.45, the propulsive force generated by the caudal fin in one swing period gradually increases as the St number increases, but the range of the increasing amplitude gradually becomes smaller. In addition, when the phase difference is in the range of 50°~60°, the propulsion of the caudal fin is relatively large and this is a more optimized motion mode
3. For hydrodynamic studies of double caudal fins, changing the angle of attack of the double caudal fins will produce different wake flow field structures. A reasonable use of the wake vortex generated by the front caudal fin will help the rear caudal fin to reduce the resistance and generate a propulsive force more effectively
4. By studying the motion law of the tail of swordfish, the motion fitting of the tail swing was carried out by using the link mechanism which was widely used in machinery. By calculating the thrust of the simplified tail swing model, the principle of the thrust generated by the caudal fin and caudal peduncle in the process of propulsion was analyzed. By controlling the angle of the steering gear, the fish body wave was fitted to the fish tail motion. In this way, the biomimetic motion mechanism of the caudal fin was preliminarily studied

## 6. Future Work

Based on the present study on theoretical calculation analysis of the caudal fin, the authors will build an experimental platform for the experimental analysis of the swing angle of the caudal fin by using the linkage mechanism, which further verifies the correctness of our simulation results in the future work.

## Figures and Tables

**Table 1:** Boundary condition setting.

	Type	Value
Inlet	Velocity-inlet	0 m/s
Outlet	Pressure-outlet	0 Pa
Upper and lower boundary	Wall	—
Caudal fin model	Wall	—

## Data Availability

The data used to support the findings of this study are available from the corresponding author upon request.
